# The links between chronic obstructive pulmonary disease and comorbid depressive symptoms: role of IL-2 and IFN-γ

**DOI:** 10.1007/s10238-015-0391-0

**Published:** 2015-09-24

**Authors:** Joanna Rybka, S. Mechiel Korte, Małgorzata Czajkowska-Malinowska, Małgorzata Wiese, Kornelia Kędziora-Kornatowska, Józef Kędziora

**Affiliations:** 1 Department and Clinic of Geriatrics, Collegium Medicum UMK in Bydgoszcz, M. Curie Skłodowska St. 9, 85-094 Bydgoszcz, Poland; 2 Division of Pharmacology, Utrecht Institute for Pharmaceutical Sciences (UIPS), Utrecht University, Utrecht, The Netherlands; 3 Department of Lung Diseases and Respiratory Failure, Kujawsko–Pomorskie Pneumonology Centre, Bydgoszcz, Poland; 4 Department of Immunology (Faculty of Pharmacy), Collegium Medicum UMK in Bydgoszcz, Bydgoszcz, Poland; 5 Collegium Medicum UMK in Bydgoszcz, Bydgoszcz, Poland

**Keywords:** COPD, Depression, IL-6, IL-2, IFN-γ, T_reg_, Neopterin

## Abstract

Depression is highly prevalent in COPD patients, and both diseases are believed to be associated with inflammation. The aim of this study was to elucidate the role of the immune system alterations in pathogenesis of depression in COPD patients. Blood was collected from patients diagnosed with chronic obstructive pulmonary disease and comorbid depressive symptoms [COPD + DS, (*N* = 13)], from individuals with either COPD (*N* = 16) or recurrent depressive disorder (rDD) alone (*N* = 15), and from healthy controls (*N* = 19). Surface phenotype expression of T regulatory and T effector cells was analyzed with a flow cytometry, and IL-2, IL-6, IL-8, IFN-γ, IL-17, and neopterin were detected with ELISA. We demonstrated that COPD, depression, and COPD with comorbid depression are associated with increased IL-6 levels when compared with healthy controls 42.2 ± 1.87, 40.9 ± 2.12, 41.7 ± 1.31, and 33.2 ± 1.23 pg/ml, respectively (*p* < 0.05). A significant increase in neopterin levels was observed both in rDD and COPD patients when compared with controls (15.69 ± 0.095, 13.98 ± 0.887 vs. 9.22 ± 0.466 nmol/l, *p* < 0.001 and *p* < 0.05, respectively). Concentrations of IFN-γ were significantly increased in COPD + DS patients when compared with controls (24.3 ± 1.49 and 17.8 ± 0.70 pg/ml, respectively, *p* < 0.05). IL-2 levels were highest in COPD + DS (3.20 ± 0.389 pg/ml) and differed significantly when this group was compared with controls (2.20 ± 0.184 pg/ml), *p* ≤ 0.05). In this study, we demonstrated for the first time that depressive symptoms in COPD patients may be related to inflammatory state as confirmed by increased levels of IL-6 both in COPD and depression and also in COPD with comorbid depressive symptoms, despite the fact that the patients were treated with anti-inflammatory drugs and/or antidepressants. We also identified IFN-γ and IL-2 as putative inflammatory agents associated with depressive symptoms in COPD patients. Prospective studies will need to confirm whether measuring IL-2 and IFN-γ can identify COPD patients at risk of depression. These findings suggest that T helper cell 1-derived cellular immune activation may play significant role in developing depressive symptoms in COPD patients.

## Introduction

Mounting evidence underscores the important role of immunological and immunopathological processes in depression. Enhanced secretion of pro-inflammatory cytokines affects the brain and elicits various symptoms of depression [1, 2]. Alterations in immune function secondary to increased psychological stress also mediate immune-based diseases such as asthma and atopy [3]. The high comorbidity of depression with conditions associated with immune vulnerability strongly supports the involvement of the immune system in precipitating depressive symptoms. Indeed, we and others have already provided evidence for altered immune response and related inflammation in depressed patients [4, 5, 6].

In order to better understand the pathogenesis of major depression, we choose a model of comorbid depression and investigated the role of the immune system and inflammation in depression, chronic obstructive pulmonary disease (COPD), and comorbidity of the diseases. Of note, depression is highly prevalent in patients suffering from COPD [7, 8], and importantly, patients with COPD have a higher probability of a first episode of depression compared with control subjects [9]. Furthermore, both depression and COPD are associated with a dysregulation of the cytokine balance [3] and decreased responsiveness to anti-inflammatory effects of glucocorticoids [10], which may increase the subsequent risk of poor functioning of the immune system and lead to the chronic inflammation [11].

The activity of the immune response is coordinated by T helper (Th) cells. Growing body of evidence suggests that adaptive immune responses and related T cell activation play an important role in the pathogenesis and progression of COPD and depression. Activated T cells can cause a variety of tissue injuries that typify COPD by direct cytopathic effects, elaboration of diverse proinflammatory and deleterious mediators, and/or recruitment and activation of other immune effector cells [12]. Therefore, activation of pulmonary and systemic immune response should be considered a plausible risk factor for development and progression of chronic inflammation, and this can trigger other pathologies comorbid to COPD including depression [13]. Alike in COPD and depression, impaired T cell function may directly contribute to development of the disease, and this link was based on the notion that T cells may subserve neuroprotective and anti-inflammatory functions during stress and inflammation [14]. From a functional perspective, Th cells can also be classified into regulatory cells (nT_reg_) that show immunosuppressive activity and into T_effector_ cells that combat infectious pathogens. Data indicate that T regulatory cells may also play a role in depression through downregulation of chronic inflammatory responses [15]. Altogether, these findings are calling for more research to understand the complex nature of immune regulation and inflammatory response in specific conditions such as depression and COPD, including the role of T cells which orchestrate and regulate physiological adaptive immune responses and manipulate immune system in pathologic conditions [16].

In line with the above, the aim of this study was to elucidate the relationship between parameters of cell-mediated immune response and depression in COPD patients. In order to determine common and distinct features of immune response in COPD and depression, we analyzed the systemic immune response in the blood of patients from the following groups: COPD, recurrent depressive disorder (rDD), and COPD with comorbid depressive symptoms (COPD + DS). Using this approach, we provide the findings important for understanding the role of immune activation for the risk of depression in COPD patients. The specific parameters analyzed in our study were balanced between T lymphocytes subpopulations CD4^+^CD25^high^CD127^low^ and CD4^+^CD25^−^CD127^−^. The former has been annotated as T_regs_ and play a critical role in the maintenance of peripheral tolerance and immune homeostasis against self-antigens owing to their capacity to reduce the activation and expression of conventional T cells by suppressing their biological activities like proliferation and blocking the production of proinflammatory cytokines [17]. Inflammatory response was assessed by means of measuring peripheral levels of cytokines and neopterin. Specifically, we measured concentration of interferon gamma (IFN-γ) which is a pleiotrophic cytokine with immunomodulatory effects on a variety of immune cells [18]. In macrophages, IFN-γ stimulates the release of large amounts of neopterin, a secondary marker of T helper cell 1-derived cellular immune activation [19, 20, 21]. We also measured levels of interleukin 2 (IL-2) that acts in synergy with IFN-γ and contributes to the production of IFN-γ by human T cells [22]. Another cytokine measured in our study was interleukin 6 (IL-6) which is a multifunctional cytokine, and its signaling pathways converge of the pathways of IFN-γ [23]. We also assessed the level of interleukin 17A (IL-17A) which can be produced by various subtypes of T cells and activate other cells to produce IL-6 and interleukin 8 (IL-8), the latter also measured in this study [21, 23]. Although IL-17 is important pro-inflammatory cytokine and its role have been extensively studied in airway inflammation [21, 24, 25], only few studies have focused on the role of IL-17 in major depression and providing inconclusive results [26]. Altogether, in this study we aimed to find out possible differences in the profiles of cellular immune activation mediators between patients with COPD and depression and delineate the relationship between these profiles and depressive mood in COPD.

## Methods

### Subjects

A group of 44 age- and sex-matched patients diagnosed and currently treated either for COPD (*N* = 29) or rDD (*N* = 15) both diagnosed according to ICD-10 criteria, and healthy controls (*N* = 19) were recruited from the Kujawsko--Pomorskie Pneumonology Centre, Psychoneurology of the Elderly Center, *Sue Ryder* Home and Department and Clinic of Geriatrics, Collegium Medicum UMK (Bydgoszcz, Poland). Among the patients with COPD, we distinguished two subgroups either with COPD alone (*N* = 16) or with COPD with comorbid depressive symptoms, COPD + DS (*N* = 13). The 21-item Beck Depression Inventory (BDI), cut–off >13 [27], was used to assess severity of depressive symptoms a week before their enrollment in the study.

All subjects were evaluated by standard physical examination and routine clinical laboratory tests. Both patients and controls with other psychiatric diagnoses, concerning axes I and II disorders and other medical illnesses (diabetes, endocrine disorders, hepatitis, cancer or chronic infections), were excluded from the study. All patients were on their usual medication at the time of blood collection. All COPD patients were treated with beta-2-mimetic, cholinolytic, theophylline, and 42 % additionally received inhaled steroids. Among depressed patients, 60 % were treated with SSRI, 35 % with SNRI, and 15 % with TCA. Table 1 shows the demographic and clinical characteristics of the controls and patients. All the patients and control subjects were native, unrelated inhabitants of the central Poland. Written informed consent was obtained from all the participants of the study. The study was approved by the Nicolaus Copernicus University in Toruń Human Ethics Committee, and it was conducted under the tenets of the Declaration of Helsinki.

**Table 1:** Demographic and clinical characteristics of patients and controls

Parameter	C	rDD	COPD	COPD ± DS	*p* value
Number of subjects	19	15	16	13	
Age (year)	62.3 ± 2.84	59.7 ± 1.91	61.9 ± 1.19	63.1 ± 1.37	0.7258
BDI	4.2 ± 1.39	17.7 ± 1.12	6.4 ± 0.71	19.6 ± 2.53	<0.0001
Cortisol (μ/dL)	21.8 ± 0.39	24.3 ± 0.38	22.6 ± 0.95	23.7 ± 0.32	0.014
C-reactive protein sCRP (mg/l)	1.2 ± 1.14	2.2 ± 0.30	2.4 ± 0.46	1.78 ± 0.07	0.641
Body mass index (kg/m^2^)	20.9 ± 1.92	24.8 ± 0.94	21.9 ± 1.79	23.9 ± 1.84	0.996
Systolic blood pressure (mmHg)	119.1 ± 6.21	124.7 ± 6.22	129.3 ± 4.87	125.1 ± 5.08	0.627
Diastolic blood pressure (mmHg)	61.3 ± 7.18	66.9 ± 5.55	76.6 ± 6.25	68.7 ± 6.92	0.404
Plasma glucose (mg %)	93.8 ± 9.11	105.0 ± 5.78	99.4 ± 6.64	91.7 ± 3.65	0.584
Total cholesterol (mg %)	191.8 ± 11.12	195.1 ± 10.07	210.2 ± 9.64	217.0 ± 15.81	0.377
Triglycerides (mg %)	101.7 ± 12.60	107.5 ± 7.61	110.5 ± 13.12	92.1 ± 8.24	0.711
WBC (G/l)	6.4 ± 0.63	6.9 ± 0.27	7.7 ± 0.34	7.2 ± 0.36	0.220

Values are expressed as mean ± SEM

*rDD* recurrent depressive disorders patients, *COPD* chronic obstructive pulmonary disease patients, *COPD* + *DS* chronic obstructive pulmonary disease with comorbid depressive symptoms, *C* control group subjects

### Blood samples

Venous EDTA anticoagulated and heparinized fasting blood samples were collected in the morning (08:00 am). One milliliter of whole EDTA blood was used for cytometric analysis of T_reg_ and T_effector_ cells. Remaining blood was centrifuged to obtain plasma and serum which was aliquoted and immediately stored at −80 °C for further analyses of cytokine and cortisol concentrations.

### Cell surface phenotype expression

The cells were labeled with FITC (fluorescein isothiocyanate), PE-Cy7 (phycoerythrin-Cy7), and Alexa Fluor^®^ 647-conjugated mouse anti-human monoclonal antibodies (MAb) (mIgG1, Becton–Dickinson), specific for different cell membrane receptors of lymphocytes surface. The FITC-labeled MAbs (Becton–Dickinson) involved: anti-CD4 (mIgG1, SK3). The PE-Cy7-labeled MAbs involved: anti-CD25 (mIgG, 2A3). The Alexa Fluor^®^ 647 (Becton–Dickinson) involved: anti-CD127 (mIgG1, HIL-7R-M21). Isotypic control was used for every prepare staining. The labeling was carried out following the manufacturer’s instructions in accordance with the stain-and-then-lyse procedure. Briefly, 20 μl of MAb was added to 100 μl of peripheral blood in accordance with staining panel. The samples were incubated for 15–20 min in dark conditions at room temperature (RT). Subsequently, 2 ml of lysing solution (Becton–Dickinson) was added to each sample and incubated for 15–20 min in dark at RT. Then, 1 ml of cold (2–8 °C) phosphate-buffered saline (PBS) was added to each sample and centrifuged (300×*g*) for 10 min at 4 °C. After removing the supernatant, 2 ml of PBS (4 °C) was added to the cellular sediment and centrifuged (300×*g*) for 10 min at 4 °C. After removing the supernatant, 200 μl of PBS (2–8 °C) was added to the sediment, and the cells were analyzed in FACSCanto II flow cytometer (Becton–Dickinson). Flow cytometry acquisition and analyses were performed on at least 100,000 acquired T lymphocytes. Lymphocytes were identified by light scatter profile and CD4-positive expression. Obtained cytometric data was analyzed using FlowJo version 7.6.1 software (Tree Star). CD4^+^ cells subpopulation of CD127^low^ CD25^high^ was identify as the natural T regulatory cells (nT_reg_), and the fraction of CD25^−^CD127^−^ was identified as T_effector_ cells.

### Enzyme-linked immunosorbent assay (ELISA) detection for IL-2, IL-6, IL-8, IFN-γ, TNF-α, IL-17, and neopterin

The concentrations of IL-2, IL-6, IL-8, and IL-17 in peripheral serum of patients and healthy controls were tested with the human ELISA kits (Diaclone ELISA, Gen-Probe). The concentrations of IFN-γ were determined using the human ELISA kits BD OptEIA™ (BD Biosciences). The concentrations of neopterin were analyzed using human ELISA kits (Labor Diagnostika Nord GmbH & Co. KG). Optical density (OD) of the cytokines and neopterin were read by a microplate reader. The means of the available duplicate sample values were used for all kits.

### Statistical analysis

The results of the measurements were analyzed by means of descriptive statistics (median, mean, and standard error of the mean). Additionally, a comparison between the groups was conducted on the basis of nonparametric statistics (Kolmogorov–Smirnov test, not shown) since the dataset could not be assumed to be normally distributed. Kruskal–Wallis nonparametric ANOVA and the Spearman’s rank correlation coefficient were calculated to find differences or correlation, respectively. We used nonparametric tests considering that small sample size did not provide enough data to discriminate between Gaussian and nonGaussian distributions, increasing the risk of inaccurate P value when running parametric tests.

Parameter associations with age, sex, BMI, systolic, and diastolic blood pressure, plasma glucose, plasma total cholesterol, triglycerides, and class of drugs were tested using regression analysis. None of these clinical characteristics had significant effect on analyzed parameters. *Statistica version 9* software was used for statistical analysis. Differences were considered significant if the two-sided *P* value was 0.05 or less.

## Results

### Cytokines

#### IL-2

**Fig. 1:**
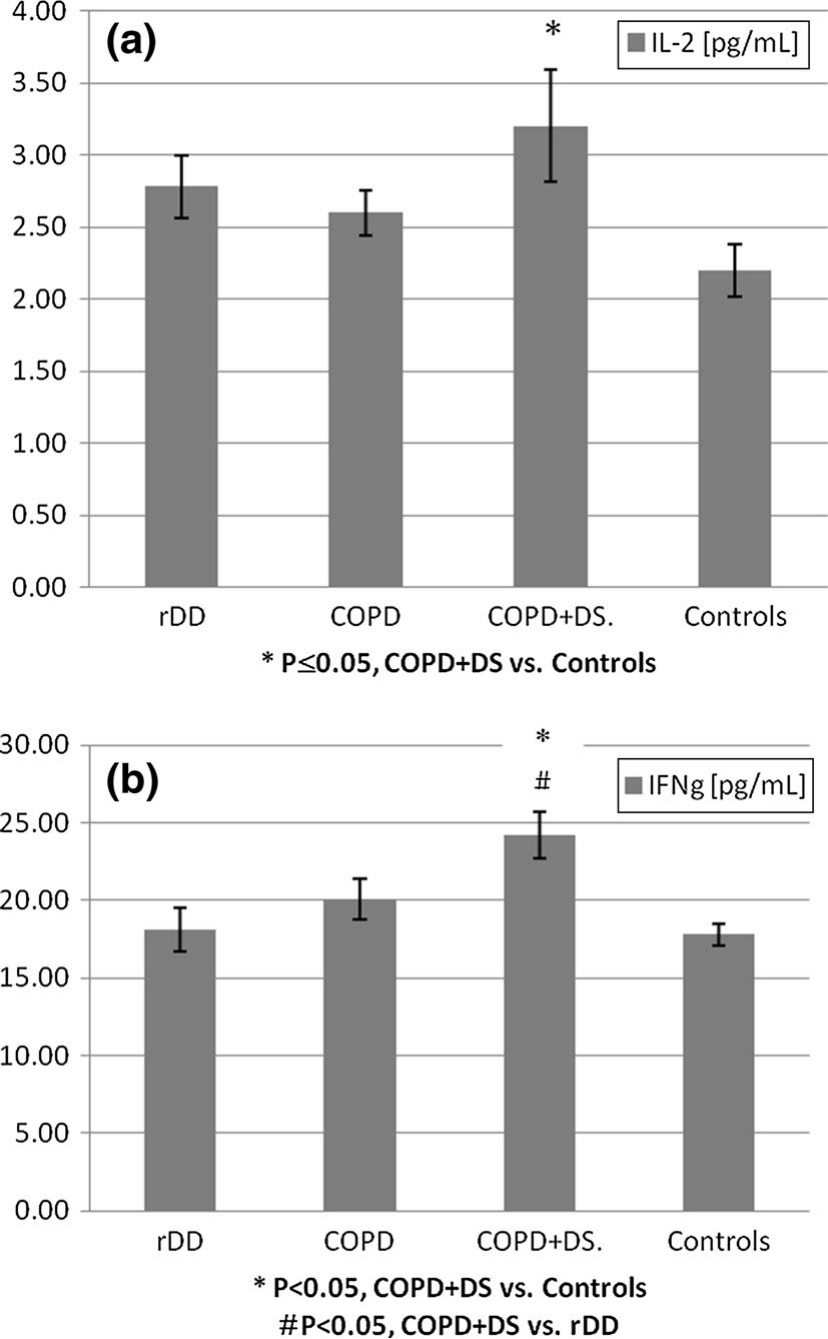
Change in plasma levels of IL-2 and IFN-γ in patients with recurrent depressive disorder (rDD), with chronic obstructive pulmonary disease with or without depressive symptoms (COPD + DS and COPD, respectively), and in normal controls. *Error bars* indicate SEM. *rDD* recurrent depressive disorder patients, *COPD* chronic obstructive pulmonary disease patients, COPD + DS chronic obstructive pulmonary disease with comorbid depressive symptoms, *Controls* control group subjects, IL-2 Interleukin 2, IFN-γ Interferon gamma

IL-2 levels were highest in COPD + DS (3.20 ± 0.389 pg/ml) and differed significantly when this group was compared with controls (2.20 ± 0.184 pg/ml), *p* ≤ 0.05). There were no significant differences in IL-2 levels between the three groups of patients namely COPD, rDD, and COPD + DS (Fig. 1a).

#### IFN-γ

Concentrations of IFN-γ were significantly increased in COPD + DS patients when compared with controls (24.3 ± 1.49 and 17.8 ± 0.70 pg/ml, respectively, *p* < 0.05) and also when compare to rDD patients (18.1 ± 1.43 pg/ml, *p* < 0.05),(Fig. 1b).

#### IL-6

**Fig. 2:**
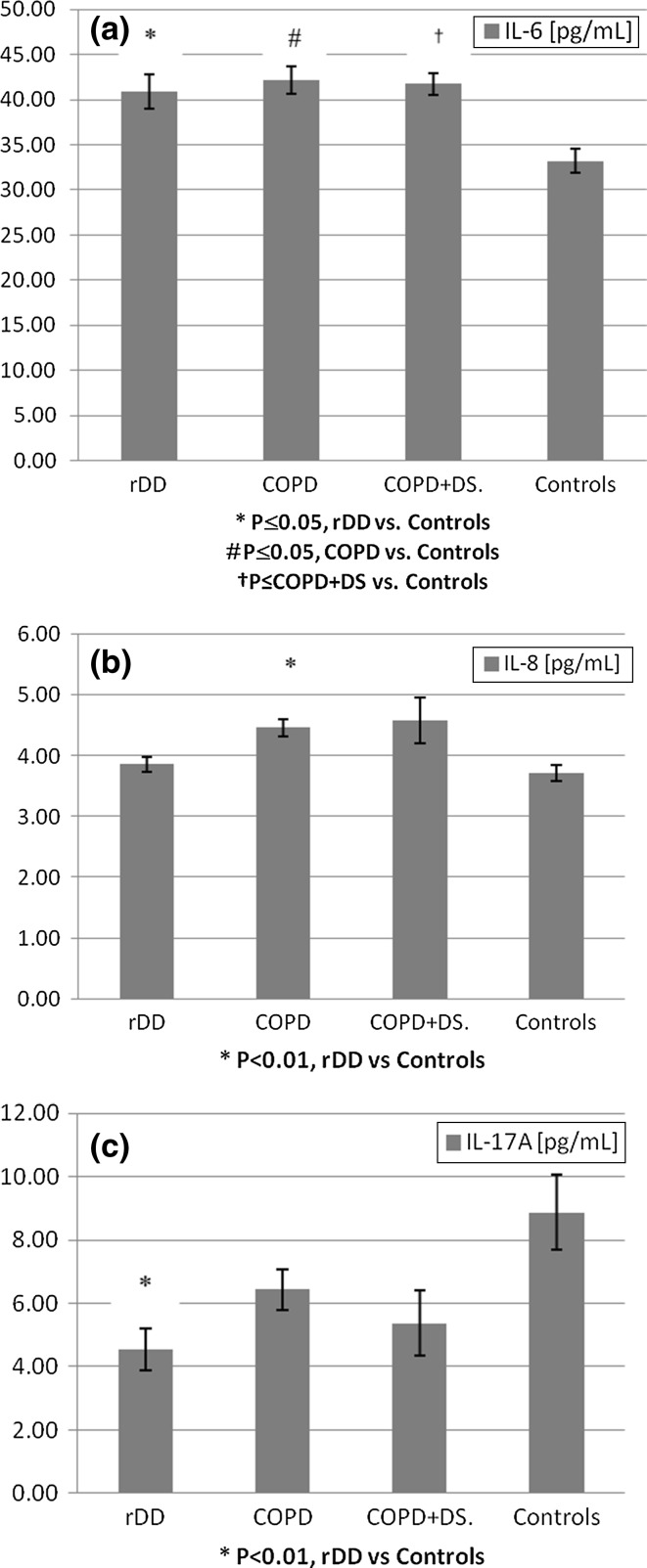
Change in plasma levels of IL-6, IL-8, and IL-17A in patients with recurrent depressive disorder (rDD), with chronic obstructive pulmonary disease with or without depressive symptoms (COPD + D and COPD, respectively), and in normal controls. *Error bars* indicate SEM. *rDD* recurrent depressive disorders patients, *COPD* chronic obstructive pulmonary disease patients, COPD + DS chronic obstructive pulmonary disease with comorbid depressive symptoms, *Controls* control group subjects, *IL*-*6* Interleukin 6, *IL-8* Interleukin 8, *IL-17A* Interleukin 17A

Concentrations of IL-6 were significantly increased in rDD, COPD, and COPD + DS groups when compared with controls: 40.9 ± 2.12 pg/ml (*p* < 0.05); 42.2 ± 1.87 pg/ml (*p* < 0.05); 41.7 ± 1.31 (*p* < 0.05) versus 33.2 ± 1.23 pg/ml, respectively, (Fig. 2a).

#### IL-8

In COPD group, IL-8 concentrations were significantly above that observed in controls (4.46 ± 0.141 vs. 3.71 ± 0.132 pg/ml, respectively, *p* < 0.01 (Fig. 2b).

#### IL-17a

We observed significantly decreased levels of IL-17A in depressed patients (rDD) when compared with controls (4.54 ± 0.670 and 8.87 ± 1.182 pg/ml, respectively, *p* < 0.01). IL-17A levels in COPD and COPD + DS were lower than in controls but did not vary significantly between patients (Fig. 2c).

#### T_regs_

**Fig. 3:**
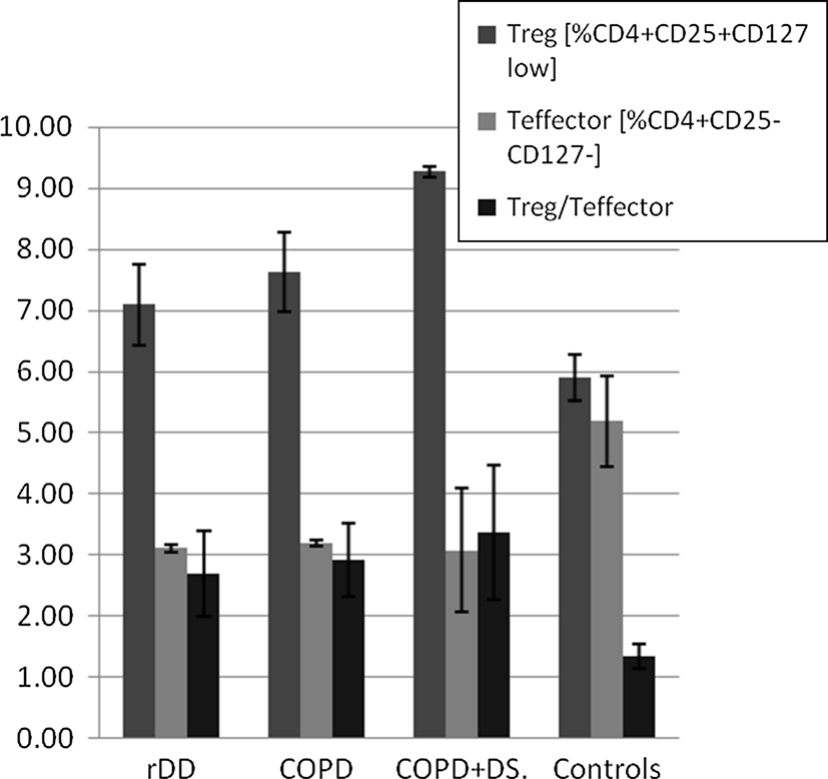
Percentage of CD4^+^CD25^+^CD127 ^low^ T_reg_ and CD4^+^CD25^−^CD127^−^ T_effector_ cells in patients with recurrent depressive disorder (rDD), with chronic obstructive pulmonary disease with or without depressive symptoms (COPD + DS and COPD, respectively), and in normal controls. *Error bars* indicate SEM. *rDD* recurrent depressive disorders patients, *COPD* chronic obstructive pulmonary disease patients, COPD + DS chronic obstructive pulmonary disease with comorbid depressive symptoms, *Controls* control group subjects, T_reg_ regulatory T cell, T_effector_ effector T cell

We also sought to analyze the balance between CD4^+^CD25^high^CD127^low^ and CD4^+^CD25^−^CD127^−^ subpopulations of T lymphocytes. The former have been recognized for their suppressive capacity and annotated as T_regs_ and the later do not exert such an activity and are considered as effector T cells. We observed differences in the proportions of the aforementioned subpopulations of T cells in all patient groups (depressed, COPD and COPD + DS) with a tendency for increased T_reg_ and decreased T_effector_ percentages when compared with controls (Fig. 3).

#### Neopterin

To further investigate the inflammatory status of the patients, we also assessed cellular immune activation by measuring serum neopterin levels. Significant increase in neopterin levels was observed both in rDD and COPD patients when compared with controls (*p* < 0.001 and *p* < 0.05, respectively). The mean concentrations of neopterin were 15.69 ± 0.095 nmol/l in rDD patients, 13.98 ± 0.887 nmol/l in COPD patients, 11.14 ± 1.176 nmol/l in COPD + DS patients, and 9.22 ± 0.0.466 nmol/l in controls.

## Discussion

In this study, we observed that both COPD and depression are associated with increased inflammation as demonstrated by increased levels of IL-6 in patients diagnosed either with COPD or with rDD and with chronic obstructive pulmonary disease with comorbid depressive symptoms (COPD + DS). Indeed, it has been already observed that both COPD and depression are associated with increased levels of IL-6 [28].

It has been suggested that because IL-6, unlike most other cytokines, is stable in the circulation, it may be involved in some of the systemic features of COPD, and in the worsening of comorbid diseases, particularly as IL-6 may contribute to impaired endothelial cell function, insulin resistance, osteoporosis, and depression [29]. In our study, we confirmed that increased IL-6 levels are indeed observed both in COPD and in depression and also when these diseases are comorbid. Interleukin (IL)-6 is produced at the site of inflammation and plays a key role in the acute phase response as defined by a variety of clinical and biological features such as the production of acute phase proteins. IL-6 in combination with its soluble receptor sIL-6R alpha dictates the transition from acute to chronic inflammation by changing the nature of leukocyte infiltrate (from polymorphonuclear neutrophils to monocyte/macrophages). In addition, IL-6 exerts stimulatory effects on T cells and B cells, thus favoring chronic inflammatory responses [30]. Therefore, the changes in IL-6 levels observed in this study may suggest that COPD and depression as well as COPD with comorbid depression are associated with chronic inflammation.

Recently, the role of IL-6 in depression was linked to increased IL-6 trans-signaling whereby IL-6 receptor signaling occurs in cells not normally expressing the IL-6 receptor. There is evidence showing that IL-6 trans-signaling is pro-inflammatory, whereas classic IL-6 signaling via the membrane-bound IL-6R is needed for regenerative or anti-inflammatory activities of the cytokine [31]. Existing body of research revealed the association between IL-6 and crucial aspects of depression including tryptophan catabolite pathway, melatonin, and neuroprogression as well as central inflammation more broadly [32, 33]. Furthermore, recently it was suggested that trans-signaling is a dominant mechanism for the pathogenic neuroinflammatory and neurodegenerative actions of interleukin 6 in the brain [34]. IL-6 also plays a role in the regulation of autoimmune processes modulated by T helper 17 cells and is associated with IL-17 production. The differential contribution of the classical and trans-signaling IL-6 pathways in cell-mediated inflammatory processes was recently demonstrated in the experiment of pharmaceutically targeting each of them using two murine models of human arthritis. The blockade of the classical IL-6 signaling resulted in decreased IL-6 and IL-17A ex vivo responses and decreased Th17 IL-17A and IL-17F mRNA gene expressions [35]. Although in our study we observed decreased levels of IL-17, there is also evidence linking high levels of IL-17 and Th17 cells to depression in animal model [36] and anxiety scores in humans [37]; however, the potential involvement of IL-17 axis in a major depression is not evident [26].

Another important aspect of IL-6 function is its ability to switch the differentiation of monocytes to macrophages [38]. Macrophages are involved in both innate and adaptive immune responses. Macrophages, particularly when activated by interferon gamma (IFN-γ) or by lipopolysaccharide (LPS), have the capacity, through the production of NO and other intermediates, to destroy the remaining microorganisms in the inflammatory loci [38]. In COPD, patients’ chronic infections are caused by LPS from the outer membrane of gram-negative bacteria. Macrophages when activated upon stimulation produce neopterin whose concentration in body fluids provides information about T helper cell 1-derived cellular immune activation [19]. Indeed, we observed significantly increased levels of neopterin in both COPD and rDD groups when compared with healthy controls and that suggests macrophage activation in both groups. Our results are in concordance with the findings of others who also found reported increased circulating neopterin both in COPD [39] and depressed patients [40].

In this study we also observed alternations that were specific for COPD + DS group and included increased levels of IFN-γ and IL-2, which further corroborates Th1-derived cellular immune activation in this group. Changes in IFN-γ and IL-2 levels and their important role for development and progression of COPD and depression have been already reported by others [28, 41].

In COPD, inflammation is associated with distinctive pattern of cytokines which are secreted by T lymphocytes. T cells in COPD are predominantly CD8^+^ (cytotoxic) cells, but CD4^+^ cells are also increased. Th1 and Tc1 subtypes are characterized by production of IFN-γ and IL-2 [28]. IFN-γ is an essential component of the host immune responses to pathogens. It has a variety of effects on the immune system, including stimulating monocytes and macrophages to secrete more interleukin 1 (IL-1), IL-6, TNF-λ, and IFN-α [42]. There is evidence that the number of IFN-γ-producing lymphocytes is increased in the lungs of COPD patients [43], IFN-γ levels are raised in the airways of COPD patients, and IFN-γ signaling is increased in the lungs of COPD patients [44]. IL-2 promotes the proliferation of T cells following primary activation with antigen by binding to its high affinity receptor (IL-2R). IL-2R signals may promote cell survival, effector function, and apoptosis [45]. There is some evidence suggesting that frequency of IL-2 productions by CD8 T cells tends to be greater among COPD patients when compared with control subjects. Furthermore, COPD severity has been shown to be directly correlated with CD4 production of IL-2 [46].

There is substantial evidence showing that IFN-γ and IL-2 play important role also in depression. It has been observed that IFN-γ when given to humans produce symptoms of depression. INF-γ usually provokes fatigue, malaise, headache, lack of appetite, weight loss, weakness, lethargy, and decreased concentration. Severe lethargy, impaired memory, slowed responses, impaired attention, anorexia, lack of interest, and irritability are the symptoms found with most volunteers after receiving low doses of IL-2 [47]. Furthermore, lymphocytes from severely depressed subjects secreted three times more INF-γ than lymphocytes from healthy controls as reported by Maes [40]. Other clinical investigations by Maes have revealed that soluble interleukin 2 receptors (sIL-2Rs) levels also significantly increased in depressed subjects and doubled the concentrations seen in healthy people [48]. Seidel et al. [49] observed that T lymphocytes from acutely depressed patients secreted significantly more IL-2 than controls. As these patients improved, their interleukin 2 secretion returned to normal. The plausible role of IL-2 in developing depressive symptoms has been related to its neuromodulatory effect. The findings that acute or repeated injections of IL-2 induce motor and cognitive abnormalities in rodents are consistent with these clinical findings and raise the possibility that IL-2 crosses the blood–brain barrier (BBB) to alter brain function which has been confirmed by Banks and colleagues [50].

There are several implications of disturbed IFN-γ and IL-2 signaling in COPD. It has been already established that primary inflammatory insult in lungs of COPD patients can cause in turn systemic inflammation [11]. IL-2 and IFN-γ secreted by Th1 cells has been shown to regulate Th-mediated immune and allergic responses by inducing Th1 differentiation. IFN-γ secretion from natural killer (NK) cells and monocytes/macrophages is likely to be important in early host defense against infection, whereas T lymphocytes become the major source of IFN-γ in the adaptive immune response [51]. Chronic immune stimulation increase Th1-type [interferon gamma (IFN-γ)-producing] CD4^+^ cells in COPD patients [52]. IFN-γ plays a key role in Th1-type inflammation and cell-mediated cytotoxicity (reviewed [53]) and has been shown to regulate processes that have central role for resolution of inflammation [54, 55]. Therefore, increased levels of IFN-γ in depression and COPD may represent mechanism important for sustaining chronic inflammatory state in both pathologies. Our results do not explain, however, the mechanism responsible for increased production of IFN-γ in depressed or COPD patients. The existing research suggests that disturbances in IFN-γ production in response to immune activation can be related to glucocorticoid signaling [56]. Noteworthy, glucocorticoid resistance or insensitivity is a major barrier to the treatment of several common diseases associated with inflammation including chronic obstructive pulmonary disease [57] and depression [58]. Furthermore, antidepressant treatment has been shown to modulate glucocorticoid receptor [59, 60]. In line with the above, a recent study in patients with coronary heart disease (CHD) and depression found out that reduced cortisol bioavailability and attenuated glucocorticoid responsiveness due to decreased expression and sensitivity of GR may lead to insufficient glucocorticoid signaling and thus elevation of inflammation in these patients [61]. Regarding the links between inflammation and glucocorticoid response, our observation of increased IL-2 levels in depressed COPD patients may be also relevant considering the existing evidence of IL-2 induced corticosteroids insensitivity in murine T lymphocytes [62] and reduced GR binding affinity in peripheral blood mononuclear cells (PBMCs) from healthy volunteers [63]. These plausible relationships between IL-2, IFN-γ, and glucocorticoid signaling calls for further research that could provide mechanistic insight into the link between chronic inflammation, depression, and its comorbidity. Therefore, we believe that our observation of increased IFN-γ and IL-2 in COPD patients with comorbid depressive symptoms indeed revealed important link between COPD and depression which may be related to reduced corticosteroid sensitivity in immune cells and therefore decreased capacity to resolve inflammation. More research is warranted to examine whether glucocorticoid resistance increases risk of depression in COPD patients. Future studies should also determine on a mechanistic level whether the immune imbalances observed herein are mediated by alterations in Th-1 cellular differentiation, cellular trafficking, chemokine production, and/or adhesion molecule expression. Prospective studies will need to confirm whether measuring IL-2 and IFN-γ can identify COPD patients at risk of depression.

## Conclusions

In this study, we demonstrated for the first time that depressive symptoms in COPD patients may be related to inflammatory state as confirmed with increased levels of IL-6 both in COPD and depression and also in COPD with comorbid depressive symptoms. We also identified IFN-γ and IL-2 as putative inflammatory agents associated with depressive symptoms in COPD patients. These findings suggest that T helper cell 1-derived cellular immune activation may play significant role in developing depressive symptoms in COPD patients.

Presented data provide evidence that activation of cell-mediated immunity in COPD patients may be responsible for increased risk of depression in this group of patients. Therefore, our observations contribute to development of macrophage–lymphocyte model of depression.

### Limitations

The results of this study are of preliminary nature and should be interpreted with the caution. Although our analyses gave significant differences between groups, the number of patients per group needs to be increased in order to allow the extraction of sound conclusions.
